# Post-Nasal Catarrh and Diseases of the Nose Causing Deafness

**Published:** 1884-09

**Authors:** 


Post-Nasal Catarrh and Diseases of the Nose causing Deafness. *By* Edward Woakes, m. d., *Senior Aural Surgeon and Lecturer on Diseases of the Ear, London Hospital, etc. 12 mo., pp. xii, 221. Philadelphia:* P. Blakiston, Son & Co.; *Chicago:* W. T. Keener. 1884.

This little work of 220 pages treats of catarrh in its various forms, and of the ordinary chronic affections of the pharynx. These diseases, of which so much has been said by the laity, have ordinarily been ignored by the regular physician as unworthy his care, and as a result they have constituted a field upon which charlatans have entered with the greatest assurance, and most disastrous results, so far as many of their victims were concerned.

Fortunately there are now many skilled specialists who devote their time to the care of the more intractable of these affections, and our author has placed the whole subject in a lucid manner before the profession at large.

The first impression of the general practitioner, on reading the book, would probably be that it unduly magnifies the importance of the specialist, by overrating the dangers of simple affections and dwelling upon the occult results which only the laryngologist or aurist can properly treat. However, a more careful perusal will, we think, acquit the author of this charge; for it will be found that he has given specific directions for the earlier treatment of the various affections of this region, which any qualified physician may readily follow; and in pointing out the grave results which sometimes follow affections, so simple, even, as an ordinary cold, he has at the same time called attention to the prominent symptoms which indicate danger, and at the same time has done what we all demand of the specialist, *i. e.*, described the cause, effect, and the treatment of the obstinate cases, of simple diseases, and of their rare complications. Although not fully prepared to accept entirely the author's theories, we can heartily commend the book to all practitioners of medicine. E. F. I.

